# The Interactive Roles of Lipopolysaccharides and dsRNA/Viruses on Respiratory Epithelial Cells and Dendritic Cells in Allergic Respiratory Disorders: The Hygiene Hypothesis

**DOI:** 10.3390/ijms18102219

**Published:** 2017-10-23

**Authors:** Tsang-Hsiung Lin, Hsing-Hao Su, Hong-Yo Kang, Tsung-Hsien Chang

**Affiliations:** 1 Graduate Institute of Clinical Medical Sciences, College of Medicine, Chang Gung University, Kaohsiung 81362, Taiwan; joanne.chiou@msa.hinet.net; 2 Department of Otorhinolaryngology—Head & Neck Surgery, Kaohsiung Veterans General Hospital, Kaohsiung 81362, Taiwan; shsu@vghks.gov.tw; 3 Hormone Research Center and Department of Obstetrics and Gynecology, Kaohsiung Chang Gung Memorial Hospital, Kaohsiung 83301, Taiwan; 4 Department of Medical Education and Research, Kaohsiung Veterans General Hospital, Kaohsiung 81362, Taiwan; 5 Department of Medical Laboratory Science and Biotechnology, Chung Hwa University of Medical Technology, Tainan 71703, Taiwan

**Keywords:** lipopolysaccharide, double-stranded RNA, epithelial cell, dendritic cell, allergic respiratory disorder, hygiene hypothesis, rhinovirus, respiratory syncytial virus, toll-like receptor

## Abstract

The original hygiene hypothesis declares “more infections in early childhood protect against later atopy”. According to the hygiene hypothesis, the increased incidence of allergic disorders in developed countries is explained by the decrease of infections. Epithelial cells and dendritic cells play key roles in bridging the innate and adaptive immune systems. Among the various pattern-recognition receptor systems of epithelial cells and dendritic cells, including toll-like receptors (TLRs), nucleotide-binding oligomerization domain (NOD)-like receptors (NLRs) and others, TLRs are the key systems of immune response regulation. In humans, TLRs consist of TLR1 to TLR10. They regulate cellular responses through engagement with TLR ligands, e.g., lipopolysaccharides (LPS) acts through TLR4 and dsRNA acts through TLR3, but there are certain common components between these two TLR pathways. dsRNA activates epithelial cells and dendritic cells in different directions, resulting in allergy-related Th2-skewing tendency in epithelial cells, and Th1-skewing tendency in dendritic cells. The Th2-skewing effect by stimulation of dsRNA on epithelial cells could be suppressed by the presence of LPS above some threshold. When LPS level decreases, the Th2-skewing effect increases. It may be via these interrelated networks and related factors that LPS modifies the allergic responses and provides a plausible mechanism of the hygiene hypothesis. Several hygiene hypothesis-related phenomena, seemingly conflicting, are also discussed in this review, along with their proposed mechanisms.

## 1. Introduction

According to the hygiene hypothesis, the increased incidence of allergic disorders in developed countries is explained by the decrease in infections [1,2]. Several studies have shown that exposure to more LPS (lipopolysaccharide and endotoxin), a major component of the outer membrane of gram-negative bacteria, in early childhood protects against the later development of allergic disorders [3,4,5]. However, these studies were done in rural areas in Europe. An urban study examined a birth cohort in the inner-city environment, and declared that exposure to specific Firmicutes and Bacteriodetes in house dust during children’s first year of life was associated with decreased atopy and atopic wheeze. Exposure to high levels of both allergens and this subset of bacteria in infancy was also inversely related to the incidence of atopy or wheeze [6]. Another study focusing on the common cold also concluded that more runny nose episodes in infancy protected against later atopy [7]. Some questions, however, remained to be answered, especially the mechanisms of protection, the nature of protective infections, and why, after sensitization, LPS seems to exacerbate the conditions, rather than protect the organisms [8]. The mechanism by which LPS might downregulate allergic airway inflammation and subsequently suppress airway hyperreactivity was not clear, but two recently published studies proposed a plausible model that clearly elucidates the role of LPS in downregulating allergic inflammation [9,10]. In this review, we propose a model that includes four major players: LPS, dsRNA or viruses, epithelial cells and dendritic cells. This model is used to explore a possible protective mechanism based on more frequent occurrences of the common cold and more LPS exposure in early childhood (i.e., before sensitization) leading to less development of allergy.

## 2. Four Major Players in the Proposed Simplified Model of Hygiene Hypothesis: Epithelial Cells (ECs), Dendritic Cells (DCs), dsRNA and LPS

### 2.1. Epithelial Cells Play Key Roles in Bridging the Innate and Adaptive Immune System

The airway epithelium contributes significantly to the barrier function of airway tract, which has three active components: the mucociliary escalator, the intercellular apical junctional complexes and the secreted antimicrobial peptides. An impaired barrier function would increase susceptibility to infection and sensitization as well as chronic inflammation [11]. However, beyond the barrier function, which was once thought to be the only function of epithelial cells, in recent years epithelial cells were found to play key roles in bridging the innate and adaptive immune system [12,13,14,15,16]. Epithelial cells can activate DCs, B cells, and T cells, thereby stimulating their differentiation, or modifying the above effects [12]. Epithelial-derived cytokines can also activate basophils, eosinophils, mast cells and nuocytes [17].

### 2.2. DCs Interact Closely with ECs to Orchestrate the Immune Responses 

DCs are responsible for initiating all antigen-specific immune responses. They capture and process antigens, express lymphocyte co-stimulatory molecules, regulate the functions of B and T lymphocytes and secrete cytokines to initiate immune responses. In addition, they are also responsible for inducing tolerance of T cells to innate antigens. Before recognition of the pivotal role of ECs in immune response, most researches emphasized the DCs [18,19,20]. Allergens, microbial compounds and various environmental and genetic risk factors for allergic disorders, however, often interfere with the immune functions of airway ECs and DCs [17,21,22]. At the 2014 International DC Symposium, at least 28 different DC subsets were described using various surface markers and nomenclature systems in distinct species [23]. However, these numerous species are generally classified into type 1 conventional DC (cCD1), type2 conventional DC (cCD2) and plasmacytoid DC (pDC) [16]. Conventional DCs, also referred to as myeloid DCs (mDCs) [24], play important roles in the pathogenesis of allergic airway inflammation. By contrast, pDCs are related to immune tolerance, and host defense against viral infections at the mucosal site, thereby modulating the extent of inflammation and tissue damage [24]. Although DCs cultured in vitro from monocytes, called moDCs, do not show the same behavior or capability as their ex vivo isolated counterpart, they are often used for research for their easier availability [23,25].

### 2.3. Most Bridging Effects Start from Activation via TLRs and Other Receptors of Epithelial Cells and DCs

Among the various pattern-recognition receptor systems of ECs and DCs, including toll-like receptors (TLRs) and NOD-like receptors (NLRs) and others, which could respond to the pathogen-associated molecular pattern (PAMP) and the danger-associated molecular pattern (DAMP), TLRs are the key systems. In humans, TLRs consist of TLR1 to TLR10 and could respond to various microbial products. However, this review discusses only the most relevant TLR3 and TLR4. Lipopolysaccharide (LPS) acts via TLR4 and dsRNA acts via TLR3. However, there are many common components in these two signaling pathways [26,27], which provide the mechanisms for interactive regulation. Furthermore, TLRs may cooperate with NLRs and PARs (protease-activated receptors) in mediating the immune response [28,29], which can be upregulated or down regulated by cytokines and chemokines in the context [30,31,32].

Below, we will discuss these two TLRs of ECs and DCs in the order of TLR3/ECs, TLR4/ECs, TLR3/DCs and TLR4/DCs.

### 2.4. dsRNA or Viruses Can Activate TLR3 of Respiratory Epithelial Cells and Stimulate the Production of Various Proallergic Cytokines

dsRNA or viruses can activate the TLR3 of epithelial cells (ECs) and stimulate the production of various proallergic cytokines [9], including thymic stromal lymphopoietin (TSLP), interleukin 33 (IL33) and IL25 [33]. Of all the TLR ligands tested, only polyI:C, representing dsRNA, the TLR3 ligand stimulates a high level of TSLP expression in keratinocytes and human bronchial ECs. Ligands of other TLRs induce only minimal expression of TSLP [31,32]. In the human epithelium IL33 is induced mainly by polyI:C and flagellin, the ligands to TLR3 and TLR5, respectively [34], following parasitic or viral infections, but also by other non-TLR factors, such as exposure to allergens. IL25 is induced by microflora, allergens, helminth or particle (TiO2) [35]. PolyI:C induces only modest expression of IL25 in respiratory ECs [9]. Downstream Th2 cytokines, such as IL4 and IL13, of the allergic triad consisting of TSLP, IL33 and IL25, would augment the stimulatory effect of polyI:C on ECs synergistically to produce more Th2 cytokines, thus promoting a positive feedback cycle [31].

Although some respiratory viruses such as influenza and respiratory syncytial virus (RSV) destroy the airway epithelial barrier, rhinovirus (RV) by itself does not cause cytopathology. The RV infection only disrupts the epithelial barrier function, specifically disrupting tight junctions, as well as increasing vascular leakage and mucus secretion [36]. In cultured human nasal ECs, RV infections showed decreased zona occluden-1, claudin-1, and E-cadherin levels, consistent with the effect of polyI:C and could expose basolateral epithelial receptors, where TLRs and other pattern recognition receptors (PRRs) are prominently located [37]. polyI:C activates TLR3 and induces apoptosis in cells [38]. Fortunately, LPS could suppress the pathogenic effect of polyI:C on ECs, and may protect the epithelial integrity [9]*.*

### 2.5. LPS Activate TLR4 of Respiratory Epithelial Cell, Using Bidirectional Capacity to Modulate Allergic Disorders through Multiple Pathways

LPS is composed of lipid A (a hydrophobic domain), a core oligosaccharide, and a distal polysaccharide (or O-antigen) [39]. Core and O-antigen sugars protect bacteria from antibiotics, the complement system, and other environmental stresses. Lipid A is the moiety that activates TLR4. 

Epidemiological studies have repeatedly confirmed that exposure to environmental LPS in early childhood would reduce the incidence of allergic disorders in later life [3,5,40]. Animal studies confirmed LPS exposure before or shortly after sensitization protects against the development of allergy [41], but after that critical time point LPS worsens the inflammation by attracting neutrophils and eosinophils, possibly via dendritic cells and B cells [42] (Table 1). Neutrophilia are discussed further in Section 2.5.3.

#### 2.5.1. Protective Role of LPS against Allergic Disorders

##### Focusing on the Role of LPS

The protective mechanism of LPS was not previously clear because LPS has little or no direct effect of stimulating ECs to produce cytokines [31,46] (Table 2). Although LPS cause a 10-fold increase in cytokine expression when serum is present [47], under general condition, when without other coexisting factors, ECs are relatively resistant to LPS in producing cytokines [48]. Thus, previously via what pathway LPS exerts its protective function was a confusing issue. However, recently, pretreatment of LPS was found to induce the A20 protein, which is a ubiquitin-modifying enzyme, and in this way reduce the inflammation caused by house dust mite in a mouse model [10]. Pretreated LPS also attenuates the induction of proallergic cytokines, including TSLP and IL33 in respiratory ECs stimulated with polyI:C and human parechovirus by attenuating TANK-binding kinase 1, IRF3, and NF-κB activation [9]. Thus, LPS does not directly antagonize the inflammation; instead, when pretreated before sensitization, it inhibits the effect of proallergic ligands of TLR3 and simultaneously induces A20, which is a potent NF-κB inhibitor [49]. Pretreatment of LPS was also shown to be protective against inflammation in other kinds of cells, including macrophages, cardiac myocytes [50], and neurons [51], under certain conditions (Table 2).

##### Focusing on the Role of LPS-Related Actions in Farming Households

The LPS levels of exposure for children from farming households are significantly higher than those from non-farming households, and an inverse association was also noted between the LPS exposure level and the prevalence of hay fever and atopic sensitization [3]. More than 20 studies focusing on the issue of protection conferred by farm environment exposure against atopy development revealed that the protective farm effect was related to microbial exposure [55]. An inverse relationship between exposure to LPS in the mattress dust of children and the occurrence of atopic diseases was also shown in rural environments [4]. The same pattern was noted with the diversity of microbial exposure inversely related to the risk of asthma [56]. The unstimulated peripheral blood mononuclear cells of farm children produced more IL10, IL12 and IFNγ than those of non-farm children, indicating increased spontaneous production of Th1 and regulatory cytokines. Decreased TNF responses to short-term LPS stimulation in farm-exposed children may imply tolerance [57]. The mRNA expression of Th1/Th2/Th17-associated cell markers decreased between ages of 4.5 and 6 years, and, at the age of six, regulatory T cells (Treg) were decreased with farm exposure and increased within asthmatics, compared with levels at age of 4.5, implicating a critical “time window” for Treg-mediated asthma protection via environmental exposure before age of six years [58,59]. Finally, it must be emphasized that not all farming environments protect against the development of asthma and wheeze in children, e.g., the keeping of hares and rabbits, using pressed hay and the presence of sheep were positively associated [59,60].

#### 2.5.2. The Proallergic Role of LPS

In asymptomatic one-month-old neonates colonization of the airways with one or more of the pathogens *S. pneumoniae*, *H. influenzae*, *M. catarrhalis*, was associated with increased risk of a first wheezy episode, persistent wheeze, acute exacerbation of wheeze, increased blood eosinophil counts and total IgE and increased risk of developing asthma by the age of five years. However, the study did not specify the mechanism of increased asthma risk, whether via ECs, DCs or their interaction [61].

Using irradiated chimeric mice, Hammad et al. demonstrated that TLR4 expression on lung ECs is required and sufficient for house dust mite (HDM) to activate DCs and prime for Th2 responses. Moreover, LPS binding to TLR4 of ECs in the presence of HDM led to the production of proallergic cytokines, including TSLP, GM-CSF, IL25 and IL33. Knockout of TLR4 on ECs, but not on hematopoietic cells, abolished HDM driven allergic airway inflammation. A TLR4 antagonist targeting exposed ECs suppressed the airway hyperreactive features of asthma [62]. Thus, the role of LPS-TLR4 binding in HDM-induced allergic disorders was confirmed.

#### 2.5.3. The Pro-Inflammatory Non-Allergic Role of LPS

When the allergic state was already established, further LPS application in the airway might induce a state of worsened inflammation with neutrophilia predominant with eosinophilia persistence or abolished [41,54]. The airway neutrophilia could be caused by two pathways: first, direct activation of the airway epithelial cells by LPS with production of IL8 [47], which is a strong neutrophil attractant chemokines [63] and second, indirect pathway with Th17 cells activated first, and then the released IL17 activates the airway epithelial cells to produce IL8 and other related cytokines [64]. Here we have to emphasize that normal airway epithelial cells are relatively resistant to common type LPS stimulation [47], yet they will become inflamed in the presence of blood or serum, but by contrast, not plasma [65,66], indicating some factors in blood or serum critically influence their responses. Besides, unusually high dose or even to the toxic level or sublethal dose as in Table 3 would activate another non-allergic inflammatory pathway with neutrophils predominant [67,68,69]. LPS of strong pathogens, such as *Pseudomonas aeruginosa* would cause robust airway inflammation even at a much less dose [70]. The role of LPS in pro-inflammatory or non-allergic effect is summarized in Table 3. In addition, the dual roles of LPS is discussed in more detail in Section 2.7, Section 3 and Section 4.

### 2.6. dsRNA or Many Viruses Activate TLR3 of Dendritic Cells, Thus, Induce DCs with Th1-Promoting Capacity with Some Exceptions

#### 2.6.1. dsRNA Activates the TLR3 of DCs, and Cause Them to Become DCs with Th1-Promoting Capacity

dsRNA can facilitate the development of Th1 cells even after the active period [73]. The Th1-promoting effect is mediated by IL12 and some unknown factor because anti-IL12 Abs could only partially block it [73], and IL12p40/p70-deficient mice could still mount a strong Th1 response [74]. DCs pretreated with polyI:C expand T cells with high Th1 polarity (70–90%), and this pattern is also confirmed in influenza [75].

#### 2.6.2. Respiratory Syncytial Virus (RSV) Is Probably an Exception, Which Likely Skew DC towards DC with Th2-Promoting Capacity

RSV infection is known to be associated with higher risk of later allergic disorders [76,77]. Clinical studies in RSV bronchiolitis revealed low Th1 responses with reduced IFNγ production and robust Th2 cytokines production [78]. In predisposed individuals, RSV infection cause the release of tissue alarmins and promote a cytokine microenvironment that is Th2 prone [79]. DCs, mainly myeloid DCs (mDCs), are likely to play pro-inflammatory roles in RSV infection and induce a Th2 response and may increase the later risk of developing allergic asthma [80]. The Th2-biased response is quite different from Th1 response to other respiratory viruses, such as influenza virus and adenovirus with strong IFNγ production. Upon RSV infection, mDCs mature, strongly activate naïve T cells and promote Th2 responses. In contrast, in healthy lungs, most of these cells are immature and unable to activate naïve T cells [80]. However, there remain some controversies. In Stein’s series, RSV lower respiratory infections (LRTIs) were associated with an increased infrequent and frequent wheeze by age six. Risk decreased markedly with age and was not significant by age 13. No association between RSV LRTIs and subsequent atopic status was noted. In addition, it was concluded that RSV LRTIs in early childhood is an independent risk factor for the later occurrence of wheezing up to age 11 years but not at age 13. This association is not caused by an increased risk of allergic sensitization [81]. In Kotaniemi-Syrjänen’s cohort series, RSV infection was found to be associated with a relatively low risk of later childhood asthma among young children hospitalized for wheezing. However, when compared with non-selected school-aged children, the risk of asthma for RSV-positive children is increased, though only less than four folds [82], not as high as reported by Sigurs et al. (12 folds) [76,77].

#### 2.6.3. Is Rhinovirus (RV) Another Exception?

Severe rhinovirus infection in infancy is closely related to later asthma development [83]. The risk of asthma at age six years in children who wheezed during the first three years of life with RV infection is 9.8-fold greater in comparison with those who wheezed without RV or RSV infection [84], with RSV-infected children displaying a 2.6-fold increase. However, in contrast to infancy, RV infection in non-asthmatic adult generally only causes runny nose, stuffy nose and sore throat, limiting symptoms to the upper respiratory tract only [85,86]. Whether RV infection predisposes the children to allergy or just reveals the children who already have an allergic predisposition is an issue to be resolved [87].

##### Related Mechanisms of Rhinovirus Infection

dsRNA and many virus infections generally would facilitate the development of Th1 cells via skewing DCs [73], but some strains of human rhinoviruses, e.g., HRV14, which belongs to the major group human RV (HRV), can efficiently inhibit the T cell stimulatory capacity of DCs through binding to its cellular receptor human intercellular adhesion molecule-1 (ICAM-1), inducing inhibitory cell surface receptors, and induce a promiscuous and deep anergic state in co-cultured T cells, despite high levels of MHC molecules as well as co-stimulatory molecules in in vitro study. This effect is independent of inhibitory soluble factors such as IL10 [88]. In this way, RV induces a hypoproliferative state in co-cultured T cells if they can come to contact DCs, but the Th1 predominant pattern is still preserved, only with decreased intensity. However, this study was performed by co-culturing HRV14 and DCs for more than 24 h in the laboratory. It is not certain if the circumstances could be reproduced in infected non-allergic individuals. Furthermore, even though the adaptive immune response was inhibited, the net response still was Th1, with the IFNγ level much higher than the IL4 level. In addition, another strain HRV2, belonging to a minor group, could bind to low-density lipoprotein receptor (LDLR) and stimulate much more production of IFNγ than HRV14 stimulation without causing anergy at all. Thusm both groups support the Th1 response, but maybe with different strengths [88].

##### Question: Under Normal Circumstances, Will Rhinoviruses Easily Approach DCs, as Shown in the In Vitro Study Above?

In Arruda’s series done on nasal and nasopharyngeal biopsy tissue infected by HRV, only low numbers of ciliated cells were infected by HRV. Infected non-ciliated epithelial cells were also detected in the nasopharynx, indicating that only a very small proportion of cells (usually ≤10%) in the nasal epithelium and in both ciliated and non-ciliated cells in the nasopharynx were infected [89,90]. In the lower airway epithelium, the frequency of rhinovirus-infectable cells was noted to be similar to that in the upper airway [90]. The infected epithelium revealed no cytopathic change, and in studies of both natural and experimentally-induce colds, no viral RNA can be detected in the subepithelial layer. Thus, the viral infection is confined to the epithelial layers, with the degradation product of RV possibly via lysosome or proteasome pathways activating the epithelial cells to release various cytokines, chemokines etc. and attracting inflammatory cells [91]. Another study concluded that RV viremia was rare in children with respiratory infections, with 25.0% (7/28) children viremic with asthma exacerbations, 7.7% (2/26) viremic with common cold, 4.0% (1/25) viremic with bronchiolitis, and 0% (0/9) viremic with pneumonia [92]. Further experiments should be done to elucidate the possibilities of direct stimulation of RV on DCs. At present, there is limited data because most HRV strains (major groups) do not bind to murine receptor ICAM-1, so no proper murine model of HRV can be used.

##### In Transgenic Mouse Model

In the ICAM-1 transgenic mouse model, the minor group RV-1B caused neutrophilia and lymphocytosis without detectable thymic stromal lymphopoietin (TSLP), IL4, IL13 and IL17. IFNγ production was also increased in murine lung leukocytes, thus favoring Th1 response. This study proposed that allergic airway inflammation could be exacerbated by RV-induced Th1 response [93]. However, there are approximately 150 strains of rhinovirus, and different RV strains may have different stimulating capacities in the induction of allergic disease.

##### Conclusion on the Protective or Pro-Inflammatory Role of RV

**Figure 1:**
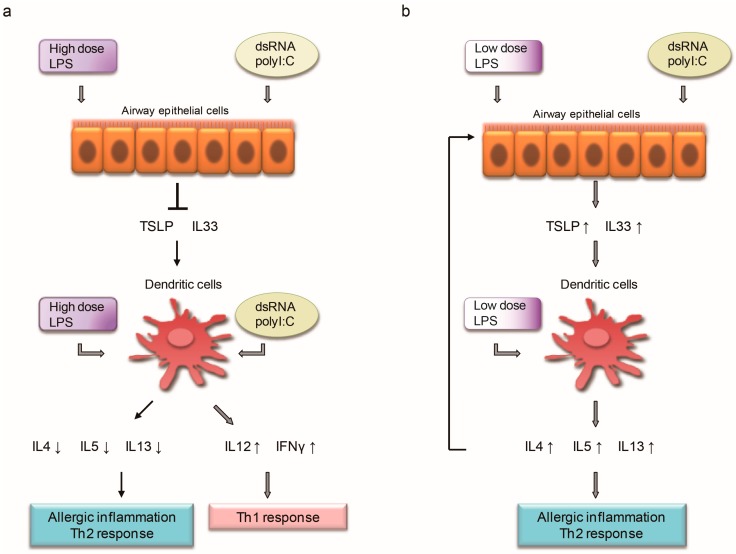
(**a**) Before sensitization, when the LPS level in the environment is high, each episode of common cold infection (dsRNA) cause no effect on epithelial cells (ECs), but stimulate dendritic cells (DCs) to skew towards Th1 prone except RSV infection, which is Th2 prone. (**b**) When the LPS level in the environment is low, each episode of common cold infection (dsRNA) then stimulate the epithelial cells to produce proallergic cytokines, such as thymic stromal lymphopoietin (TSLP) and IL33, which would facilitate T cells to produce more IL4, IL5 and IL13, and these Th2 cytokines would work synergistically with dsRNA to stimulate ECs to produce more IL33 and TSLP, forming a positive feedback cycle, and thus shaping the allergic predisposition seen clinically.

For non-sensitized individuals, whether infants or adults, RV infection causes a minimal epithelial reaction, with a minimal Th2 response, which could possibly be easily suppressed by LPS when LPS of non-pathogenic bacteria are present. RV infections then stimulate DCs to produce a minor Th1 response (Figure 1a), because most of the infections are localized to the epithelial layers, so the viral load on DCs is minor, and would not cause the severe neutrophilia and lymphocytosis as seen in mouse model due to RV-1B [93]. However, in sensitized individuals, RV infections would stimulate the ECs with junctions of epithelial layers disrupted, proallergic cytokines such as TSLP and IL33 would be released and stimulate DCs towards Th2-promoting development. In addition, more viruses may penetrate the epithelial layers and stimulate DCs towards Th1-promoting development. The net result is that both Th1- and Th2-promoting activities of DCs would be increased, as noted in clinical cases of the exacerbation of the asthmatic patients after RV infection [94]. More studies are necessary to prove this deduction, although it is compatible with the already published data.

### 2.7. LPS Has the Potential to Activate Immature Dendritic Cells (DC) into Mature DCs with Th1- or Th2-Promoting Capacity

LPS stimulates DCs to produce IL12 transiently and thus induces strong Th1 polarization (the so-called “active period”), but shifts to induce Th2 polarization afterwards in the “exhausted” period [45]. During the immune response, there is a dynamic regulation of the Th1/Th2 cells balance. The polarity can also be influenced by the dose of superantigen toxic shock syndrome toxin-2 with a high dosage (10 ng/mL) favoring Th1 skewing, and a low dosage (0.1 ng/mL) favoring Th2 skewing. Exogenous IL12 or IL4 is the third factor that would move the total profile towards Th1 or Th2 polarity respectively [45]. In this model, the predominance of IL12 or IL4 determines the central axis of Th1/Th2 polarity, with minor modifications by the high/low dose of antigens and active/exhausted factors. Therefore, active DCs with high dose antigen in the presence of IL12 would yield the maximal Th1 response, and exhausted DCs with low dose antigen in the presence of IL4 would yield the maximal Th2 response. In another mouse model in which ovalbumin (OVA) was used as allergen at fixed dose, the concomitant use of 100 μg (high dose) or 0.1 μg LPS in sensitizing period led to Th1 and Th2 responses later respectively. Again the dose of LPS determines the final Th1/Th2 skewing pattern [43]. When the allergen was changed to HDM, in a mouse model, increasing doses of LPS (0.001–10 μg) dose-dependently inhibited HDM-induced eosinophil recruitment and the production of Th2 cytokines in the lungs, revealing a shift toward Th1 inflammation with predominant neutrophilia [95]. However, the local switching of the airway inflammation from eosinophilia to neutrophilia did not quench the airway inflammation; instead, airway hyperreactivity was increased [54] in accordance with the clinical observation [96] (Table 1).

## 3. The Protective Role and Mechanism of LPS: Pre-Exposure to LPS Protects the Respiratory Epithelial Cells and Downregulates the Effect of dsRNA or Allergen in Producing Proallergic Cytokines, Indicating a Delicate Cross-Regulation Mechanism Exists between dsRNA (TLR3 Pathway) or Allergen and LPS (TLR4 Pathway), at Least at Epithelial Level

### 3.1. Pretreatment with Lps Attenuates Induction of Proallergic Cytokines, TSLP and IL33 in Respiratory Epithelial Cells Stimulated with polyI:C and Human Parechovirus.

In H292 cells, polyI:C activated the TLR3 signaling pathway with activation of the transcription factors IRF3 and NF-κB p65/50 by I kappa B kinase (IKK) and IKK-related kinases, such as TBK-1, IKKϵ, IKKα, and IKKβ. IRF3 phosphorylation was detected at 3 h after stimulation [26]. Phospho-TBK1, -IRF3, and -NF-κB p65 (Ser456 and Ser536) was markedly increased at 12 h. The protein level of NF-κB p65 and IκBα degradation was increased with downstream production of allergic cytokines, such as TSLP and IL33 increased as well [9]. The polyI:C-induced IRF3 phosphorylation was inhibited by pretreatment of high dose (30 μg/mL) LPS, but by contrast, lower dose of LPS (0.3 μg/mL) enhanced polyI:C-mediated IRF3 phosphorylation as compared with polyI:C stimulation alone. High dose LPS pretreatment significantly decreased IRF3 phosphorylation induced by polyI:C stimulation. The total NF-κB p65 level was not significantly changed by LPS pretreatment, but 3 and 30 μg/mL LPS treatment inhibited the polyI:C-induced IκBα degradation, which suggests that the polyI:C-mediated activation of the NF-κB pathway was downregulated with high dose LPS [9].

### 3.2. Pretreatment with LPS Protects against Allergy through A20 Induction in Lung Epithelial Cells

After pretreatment with LPS in the mouse model, the lung levels of granulocyte macrophage colony-stimulating factor (GM-CSF, the maturation factor of recruited lung DCs) and CCL20 protein (the chemokine of attracting DCs [62]) induced by house dust mites were reduced through A20 induction. A20 is a negative regulator induced by allergen stimulation via NF-κB activation to avoid deleterious effect due to overstimulation and maintains homeostasis [97]. IL33 mRNA was noted to be decreased with Th2 cytokine levels also downregulated, including IL5 and IL13 [10].

### 3.3. Pretreatment with E. coli in Mice Models Protects against Allergy via Two Pathways

Pretreatment with *E. coli* in mice models via intranasal inoculation leads to suppression of allergic airway inflammation by recruited γδ T cells (a subset of T cells with potent cytotoxicity and interferon-γ production [98]), and dampening of DC function in the lung, thus decreasing the effectiveness of presenting antigen to effector T cells [99]. These findings may be the associated events of Section 3.1 and Section 3.2. Though Th1 and Treg responses do not play a role in the context, γδ T cells are noted for their potent cytotoxicity and interferon-γ production.

### 3.4. Pretreatment with Salmonella enterica Serovar Typhimurium Protects against Allergic Airway Inflammation in Mice

Intragastric pretreatment with *Salmonella enterica serovar typhimurium* in murine models leads to protection from induced allergic airway inflammation via expansion of a CD11b^+^ Gr1^+^ myeloid cell populations, which reduce airway inflammation by influencing Th2 cells. These groups of myeloid cells consist of macrophages, immature granulocytes, early myeloid progenitors, and DCs, and exhibit their inhibitory effect by altering GATA-3 expression and IL4 production by Th2 cells [53]. 

### 3.5. LPS Suppresses Asthma-Like Responses via Nitric Oxide Synthase (NOS2) Activity

The regulatory role of NOS2 in airway allergy was revealed. Mice were immunized with OVA on Days 0 and 7, then on Day 14, challenged intranasally with OVA/saline to induce an allergic airway response, and on Day 21, rechallenged with intranasal OVA concomitantly with either intranasal or intravenous delivery of LPS. LPS via both routes completely suppressed airway eosinophilia, but in the intranasal group, the total cell number in the bronchoalveolar fluid was not reduced. The suppression effect was lost in NOS2^−/−^ mice, indicating the LPS suppressed the allergic inflammation via NOS2 activity. However, in this experiment, LPS was given after the establishment of allergic state [54]. Thus, whether pretreatment with LPS would also work via this way must be confirmed by further study.

## 4. The Pro-Inflammatory Role of LPS: Why Does LPS Induce Inflammation, Instead of Protecting against Inflammation on Many Occasions?

Although LPS acts like a protector of respiratory epithelial cells against allergic inflammation, on many occasions, it creates difficulties by inducing a large amount of inflammatory responses, even to the extent of shock or death of the host. There are probably several reasons for this. Table 3 summarized some concepts shaped by already published studies.

### 4.1. First, the Timing of Delivering LPS

In rat models, LPS protects the animal only when it is delivered before or shortly after allergen sensitization. After that, LPS exacerbates the inflammatory responses, often with neutrophils predominant [41]. In asthmatic children, a significant correlation was found between levels of LPS and airway neutrophils in bronchoalveolar lavage [100].

### 4.2. Second, the Dose of LPS Delivered

In a mouse model, 100 ng LPS every other day for two weeks before house dust mite (HDM) sensitization or 1 μg two weeks before HDM sensitization suppress all of the key asthma feature [10]. In contrast, when 8 mL of 100 μg/mL LPS suspension was nebulized and delivered in the mouse model, inhalation of LPS results in acute neutrophilic inflammation of the distal air spaces of the lungs [48]. Another mice experiment indicated that allergen sensitization with low dose LPS (0.1 μg) and OVA induced type 2 responses with airway hyperresponsiveness, eosinophilic inflammation, and allergen-specific IgE up-regulation, but, again, sensitization with high dose LPS (10 μg) and OVA induced asthma phenotypes with noneosinophilic airway inflammation [43]. In an epithelial cell line model, 30 μg/mL LPS in culture medium attenuates induction of proallergic cytokines, TSLP and IL33 in response to polyI:C or human parechovirus, but 0.3 μg/mL LPS enhanced the induction effect with increased phosphorylation of IRF3 and decreased inhibitors of NF-κB, the IκBα on the contrary [9].

### 4.3. Third, the Monocytes/Macrophage or Dendritic Cells Which Are Also Activated by LPS

As little as 10 ng/mL LPS would induce the release of inflammatory and chemotactic chemokines from these cells [101], in contrast to the 10,000 ng/mL LPS required for activation of respiratory epithelial cells [47]. If the monocytes/ macrophage or dendritic cells were activated, they would release several cytokines, such as tumor necrosis factor-α and IL1, and enhance epithelial barrier dysfunction [70].

### 4.4. Fourth, the Synergistic Effect between LPS and Environmental Cofactors

The synergistic effect between LPS and environmental cofactors, such as concomitant ozone exposure, causes worsened inflammation. In rat model, 100 μg LPS intranasally instilled cause slight neutrophilia in nasal epithelia in comparison to saline (relative value 19/14), but pretreatment with ozone before instillation of same amount of LPS cause significant neutrophilia (relative value 33/1) [102].

### 4.5. Fifth, the Resource of LPS Delivered

In the Copenhagen Birth Cohort Study, the presence of potentially pathogenic species, including *M. catarrhalis*, *H. influenzae*, or *S. pneumoniae*, in the oropharynx of one-month-old infants significantly correlates with increased risk of developing asthma in later childhood, but *S. aureus* does not [61]. Thus, it is reasonably deduced that different LPS may have a different effect, either protective or pro-inflammatory.

### 4.6. Sixth, the Presence of Serum or Whole Blood on Lung Alveolar Cells and Bronchial Epithelial Cells

The presence of serum or whole blood makes the human umbilical vein endothelial cells become very sensitive to even 0.1 ng/mL LPS [65]. When endothelial cells were incubated with whole blood or serum, they were fully activated with picomolar doses of LPS. In contrast, one thousand fold of LPS were required for the same level of activation when plasma only was used. When anti-CD14 mAbs were used, the endothelial responses to LPS in the presence of blood could be almost completely blocked. However, this enhancement effect by blood was not observed in lung epithelial cells A549 at this minimal dose [65] as reported by Pugin. However, when the LPS level was increased to 10 ng/mL, the serum would cause a much greater increase in cytokine expression in both alveolar epithelial cells (A549) and bronchial epithelial cells (BEAS-2B), perhaps through different mechanisms [47], with CD14-dependence in A549 and CD14-independence in BEAS-2B. In contrast, in the absence of serum, LPS has to reach 10,000 ng/mL (here we use ng instead of μg to emphasize the contrast) to activate respiratory epithelial cells. Currently, the significance of the presence of serum or blood in respiratory epithelial cells has not yet been fully elucidated, but the contents in inflammatory exudate may play roles in enhancing the activation of respiratory epithelial cells.

### 4.7. Seventh, the Type of Epithelial Cells Tested

As pointed out in Section 4.6, alveolar epithelial cells and bronchial epithelial cells react to LPS via different mechanisms. H292 cells, which are bronchial epithelial cells, grow well even in 30,000 ng/mL LPS up to eight days, demonstrating the relative resistance of bronchial epithelial cells [9]. Here, we preserve the nanogram (ng) scale to emphasize the difference of LPS dosage used. By contrast, if endothelial cells are tested, they are thousands or even millions times sensitive to LPS activation, with pg/mL level of LPS capable of activating endothelial cells in the presence of blood, whose concentration is only 1/1000 of the ng/mL level [66].

## 5. Proposed Mechanism Supporting Hygiene Hypothesis

### 5.1. First, Why Early Exposure to Environmental LPS, Such as Farm Dust, Would Protect against the Development of Allergic Disorder in Later Life? Two Mechanisms May Possibly Explain the Observed Phenomenon

#### 5.1.1. Pre-Exposure to LPS Attenuates the Signaling Pathway Necessary for Allergic Cytokines Production, but Spares the dsRNA/DCs Route

After a baby is exposed to more environmental LPS, which is relatively nonpathogenic, then when it later encounters a common viral infection, the dsRNA cannot activate the epithelial cells to produce allergic cytokines, such as TSLP, IL33 and IL25 [9]. Instead, it activates DCs to produce more Th1-skewing cytokines [73], and thus more Th1 cells, and more IL12. In the context of more IL12, more LPS would stimulate DCs to produce more Th1 cytokines in the presence of antigens [43,45]. Thus, each time a baby gets a common cold, it becomes more prone to Th1. Even when an allergen is present, a high dosage of LPS still favors the Th1 response to a later challenge [43] (Table 4) (Figure 1a).

#### 5.1.2. Pre-Exposure to LPS Suppresses Responsiveness of Airway Epithelial Cells via Increased Synthesis of A20

Pre-exposure of airway epithelial cells to LPS suppresses their allergic responsiveness to house dust mite (HDM) via increasing synthesis of A20, encoded by the Tnfaip3 gene. Ex vivo cultures of human bronchial epithelial cells revealed a similar result [10]. Other mechanisms may also be involved, as discussed in Section 3.3, Section 3.4 and Section 3.5.

### 5.2. Second, Why Is More Common Cold in Early Life Associated with Less Allergy in Later Life?

A German birth cohort multicenter allergy study (MAS) group confirmed that children with ≤1 episode of runny nose before the age of one year were more likely to be diagnosed as asthmatic at seven years old or to have wheeze at seven years old, compared with those with ≥2 episodes, and were more likely to be atopic before the age of five years. Similarly, having ≥1 viral infection of the herpes type in the first three years of life was inversely associated with asthma at age seven [7]. The conclusion excludes the lower respiratory infection, which instead showed a positive association with later wheeze in that study. No significant associations were found between bacterial, fungal, or gastrointestinal infections and later asthma [7]. More instances of the common cold are closely related to poorer hygiene due to more crowded living or poorer economic conditions, so on average there would be more environmental LPS. This was described in studies by Giovannangelo et al., who found that having more than four persons living in the home were consistently associated with up to 1.7-fold higher endotoxin concentrations in the mattress and floor dust [104]. Similarly, in Brazil’s study, LPS levels in day care centers and preschools were three times higher than in elementary schools [105]. Again for each episode of URI, more LPS would block the effect of dsRNA to induce allergic cytokines at epithelial level [9], and the common cold virus would stimulate the DCs towards Th1 skewing, because dsRNA stimulates both the maturation and resistance of DCs, and makes them capable of trigger naïve T cells and drives polarized Th1 responses [75] (Table 4, Figure 1a). The Tucson Children’s Respiratory Study proposed a conclusion that indirectly supports the protective effect of ‘poor hygiene’. It states that more exposure of young children to older children at home or longer stays at day care center confers more protection against the development of asthma and frequent wheezing in late childhood [106]. Maier etc. confirmed that there is a significant difference of the bacterial community structure in house dust between families with children attending day care and those without [107].

A complex question then arises: is rhinovirus beneficial or detrimental in allergic response? According to the German MAS study above, the infection of common cold virus of course is protective [7], and since around 50 to 83% of episodes of the common cold are caused by rhinoviruses [85,108], so rhinovirus should be protective. However, there is a pitfall in the study: because in that study more lower respiratory tract infections (LRTIs) before age three is positively associated with more wheeze at age seven [7]. Unfortunately except for the six months group, in which RSV accounted for most of the LRTIs, rhinovirus accounts for the largest portion of pathogens causing LRTIs alone or in combination with other viruses [82]. The other viral pathogens include RSV, parainfluenza viruses, adenoviruses and enteroviruses etc. Thus, in those children with no wheezing predisposition, according to Illis’s study, rhinovirus should be protective; but for those with wheezing tendency, rhinovirus seems to play a proallergic role. Thus, again, as with the dual role of LPS [41], rhinovirus seems to have a bidirectional capacity of enhancing or reducing the allergic tendency of children depending on their already preexisting allergic predisposition. Another indication of the effect of predisposition is that in children less than four years old, rates of asymptomatic infection range from 12 to 32% [94,109,110,111]. Readers are encouraged to refer back to Section 2.4 and Section 2.6.3 for the protective mechanism of rhinovirus.

### 5.3. Summary of the Four Major Players in Hygiene Hypothesis

Usually LPS does not directly act on epithelial cells, instead, it protects the epithelial cells against the action of dsRNA. LPS stimulates DCs to become more Th1-prone with a large dose, and more Th2-prone with a small dose, more Th1-prone in an active period, and more Th2-prone in an exhausted period, and the presence of IL12 or IL4 is the third factor, which would facilitate the Th1/Th2 skewing in the presence of antigen [45]. dsRNA differentially acts on epithelial cells and DCs, with Th2-prone on epithelial cells, and Th1-prone on DCs. Thus, before sensitization, when the LPS level in the environment is high, each episode of common cold infection (dsRNA) has no effect on ECs, and stimulate DCs to be more Th1-prone, except RSV infection, which is Th2-prone (Table 4 and Figure 1a). However, when the LPS level in the environment is low, each episode of common cold infection (dsRNA) then stimulates the epithelial cells to produce proallergic cytokines, like TSLP and IL33, which would facilitate T cells to produce more IL4, IL5 and IL13, and these Th2 cytokines would work synergistically with dsRNA to stimulate ECs to produce more IL33 and TSLP, forming a positive feedback cycle, and thus shaping the allergic predisposition seen clinically (Table 5 and Figure 1b). These four players basically regulate the key networks of immune responses related to hygiene hypothesis.

However, after sensitization has occurred, the protective effects of LPS and dsRNA may be lost, as discussed below.

## 6. Why Does the Hygiene Hypothesis Work Only before One Critical Time Point in Early life? After Allergy Is Established, Why Do the dsRNA and LPS No More Play Protective Roles?

### 6.1. Why, in Established Atopic Patients, Does Exposure to More Environmental LPS No Longer Protect Them against Allergy?

Tulic et al. proposed a mechanism, which stated that before or shortly after sensitization, LPS exposure causes the production of IFNγ and/or IL12 and drives the B cells toward IgG antibody production but inhibits class switching to IgE. Subsequently, plasma cells committed to producing IgE are already present, and these committed IgE-producing cells are resistant to regulation by IL12 and/or IFNγ. Therefore, LPS must be present before the isotype switch to IgE has occurred in order to exert its inhibitory effect [41]. In his study, LPS exposure on Days 6, 8 and 10 with challenging OVA on Day 11 resulted in more cellular influx, with increases in both neutrophils and eosinophils. Lowe performed his study on guinea pigs and concluded LPS exposure 24 h before allergen challenge diminished airway hyperreactivity (AHR) to histamine, whereas co-administered LPS prolonged the AHR [44]. The above rule seems to be applicable to DCs and in whole rat or mouse models, where LPS enhances both Th1/Th2 responses after sensitization, with Th2 response seemingly resistant to LPS treatment.

However, in Rodriguez’s study, LPS via either intranasal or intravenous route could still completely suppress airway eosinophilia [54]. The disparity may be due to the difference of experimental animals (rat vs. mice), the dose of LPS (50 μg/mL vs. 20 μg in one animal) and the pathway (inhaled by aerosol in a chamber vs. intranasally or intravenously). Similarly, in Schuijs’ study, when bronchial epithelial cells were removed from asthmatic patients via endobronchial biopsy and grown to confluence, and then cultured in air-liquid interface model, 100 ng LPS pretreatment every other day for one week still was able to reduce the production of IL1α and GM-CSF [10]. Thus, LPS seems protective still when considering respiratory epithelial cells only.

To reconcile these seemingly conflicting data, more studies are needed to elucidate the delicate and complex underlying mechanisms.

### 6.2. Why in Established Atopic Patients, Does Exposure to Viral Infections No Longer Protect Them against Allergies, but, Instead, Worsen the Allergic Disorders

TSLP production is increased in allergic individuals, and further synergistically enhanced by a combination of IL4 and dsRNA [31]. This implies that in asthmatic airway, respiratory viral infection and the recruitment of Th2 cytokine producing cells may increase the production of TSLP and amplify Th2 inflammation. When mice challenged with the already sensitized allergen, rhinovirus infection exacerbated neutrophilic, eosinophilic and lymphocytic airway inflammation, airway hyperresponsiveness, mucus secretion and production of both Th1 and Th2 cytokines [93]. Thus, the mechanisms of the synergistic interaction between virus infection and allergen exposure to increase the risk of asthma exacerbations are elucidated. Allergen exposure causes increased allergic cytokines, which would be further increased by the concomitant activation of TLR3 of epithelial cells by viral dsRNA. However, since most viral infections stimulate DCs to produce Th1 cytokines, so both Th1 and Th2 cytokines would both be increased.

In addition, in healthy humans, plasmacytoid dendritic cells (pDCs) and the IFNαβ they secrete selectively negatively regulate Th2 cytokine synthesis following RV exposure in vitro. In asthmatic patients, this important regulatory mechanism may be lost and contribute to asthma exacerbations during RV infections [114,115]. The pDCs may play key roles in healthy humans to negate the Th2-prone effect of rhinovirus. For allergic patients, the environmental LPS concentration is on average less than their normal counterpart [116], and thus confers less protection at epithelial level. Thus, when these patients contract the common cold, dsRNA would activate the epithelial cells and induce the production of allergic cytokines. In addition, increased Th2 cytokines in this context would augment the dsRNA effect in stimulating epithelial cells to produce more proallergic cytokines [31].

## 7. What Factors Initiate the Disruption of Immune Balance towards the Allergic Predisposition?

As mentioned above in Section 2.6.3, for some individuals allergic predisposition exists before rhinovirus infection. In a similar manner, some risk factors are already present before the allergy-inciting event, which make the individuals more susceptible to the development of allergic disorders. The proposed mechanisms leading to allergic predisposition include mostly genetic deficiency [10], airway and gut microbial dysbiosis (i.e., deviations from healthy microbial compositions) [79], and possibly environmental hazard factors.

### 7.1. Genetic Deficiency

Defects in TLR7/interferon regulatory factor 7 (IRF7) signaling predisposes to severe viral bronchiolitis and subsequent asthma [117,118]. Levels of plasmacytoid DCs (pDCs) during infancy were inversely correlated with childhood respiratory tract infections and wheezing up to age five years [24]. Reduced circulating pDCs during early life predisposes young children to more frequent and more severe respiratory tract viral infections and more wheezing. Recently several SNPs in the TNFAIP3 interacting protein (TNIP-1) were identified as associated with asthma, further supporting the importance of the A20 protective pathway [10]. Furthermore, common mutations in TLR4 are associated with differences in LPS responsiveness in humans, and these people are hyporesponsive to inhaled LPS [119]. Children sensitized to any allergen early in life and sensitized to inhalant allergens by the age of seven years were found to be at a significantly increased risk of being asthmatic at this age if a positive parental history of asthma or atopy was present, with the effect being strongest for maternal asthma, indicating the existence of an underlying determining genetic factor [120].

### 7.2. Microbial Dysbiosis

#### 7.2.1. Airway Microbial Dysbiosis

The microbes inhabiting the lower airways show substantial differences between asthmatics and healthy subjects. Though there are some variations, generally speaking, in asthmatics, more *Hemophilus*, *Moraxella*, *Neisseria* and *Streptococcus* spp. were found, but less *Bacteroides* [79,121]. However, once again, whether the difference of microbiome precedes and leads to the development of allergic disorders or just develops after sensitization begins needs more study to reach a firm conclusion. However, just as we have discussed above, the amount of protective LPS in the environment (which represent the amount of LPS inhaled) or the type of LPS (pathogenic or non-pathogenic) in the airways are closely related with later atopy development [61,122]. Here, we have to point out the importance of organ specificity of LPS in determining its role of protection or pro-inflammation. For example, *S. typhimurium* is an intestinal pathogen of mice, but it could protect against airway inflammation when applied before or shortly after sensitization [41].

#### 7.2.2. Gut Microbial Dysbiosis

Human guts are colonized with many trillions of bacteria, viruses and eukaryotes. Their colonization in early life plays an important role in the development of our immune system [123]. During a critical period in early life, disruption of the optimal host-commensal interactions, i.e., dysbiosis of these microbes might cause allergic disorders [124]. For example, babies born by cesarean section harbor more *Staphylococcus*, *Corynebacterium* and *Propionibacterium* than those born vaginally, but less *Lactobacillus*, *Prevotella* and *Sneathia* species [125]. The resulting change of microbiota in the intestine may lead to aberrant long-term colonization and subsequent altering of immune development [126]. Cesarean delivery is associated with a significantly increased rate of allergic disorders [127], supporting this conclusion.

A modified hygiene hypothesis suggests that an altered normal intestinal colonization pattern in infancy, which fails to induce immunological tolerance, could be responsible for the increase in allergies [128]. Two other studies concluded that bacterial diversity in the intestinal flora of infancy was inversely associated with the risk of later allergic sensitization [129,130]. Therapeutic trials with various strains of oral probiotics achieved success to varying degrees by restoring intestinal homeostasis and preventing or alleviating allergy, at least in part by interacting with the intestinal immune cells [131,132,133], although this conclusion is not agreed with by all [134,135].

Microbial products and metabolites can also affect allergic inflammation. Dietary fermentable fiber content changes the gut and lung microbiota, and the metabolized fibers consequently increase the concentration of circulating short-chain fatty acids (SCFAs), which protect against allergic lung inflammation [136], possibly via promoting peripheral regulatory T-cell generation [137].

### 7.3. Environmental Hazard Factors

Exposure to air pollutants, including higher levels of O_3_ and others, can cause acute exacerbations in those who already have allergic respiratory disorders, but its role in the initiation of new cases of asthma is not yet confirmed [138]. A cohort study of 3863 children confirmed the association between traffic-related air pollution and the development of asthma and allergies during the first eight years of life. According to the study, PM2.5 levels and NO_2_ were associated with a significant increase in incidence and prevalence of asthma [139]. In another study, infants at high familial risk for asthma were recruited and the birth year home exposures to NO, NO_2_ and PM2.5 were found to be associated with a markedly increased risk of asthma at age of seven with an odds ratio around 3 [140]. Indoor dampness and mold are also determinants for developing asthma. Chemical emissions from damp structures and surface materials may be other causal agents related to the development of asthma [141,142].

## 8. Conclusions

### 8.1. LPS Has a High Potential for Prevention Modality; However, Application of LPS as Treatment Modality Should Be Considered Cautiously

LPS, or maybe farm dust, attenuates the induction of proallergic cytokines, including TSLP, IL33, and others in respiratory epithelial cells in response to viral infection, and it does not disturb the Th1-prone effect of viruses on DCs. Thus, it should be a high potential candidate for applications of prevention modality against allergic development. However, although LPS is promising as a prevention modality against allergic disorders, the application of LPS is questionable after the establishment of allergic disorders, and may exacerbate preexisting disorders. The original Th1/Th2 balance model is not applicable since many studies have confirmed the presence of both increased Th1 and Th2 cells in asthmatic airways [143], and specifically, both Th1 and Th17 cells are crucial for the development of neutrophilic inflammation in the airways [144,145,146].

### 8.2. Limitations of the Proposed Model

In our simplified model, the role of regulatory T cells (Treg), Th17, innate lymphoid cells group 2 (ILC2) and signaling via other pathways, such as NOD-like receptors were not presented due to insufficient studies to propose an incorporating mechanism. Allergens also were not mentioned, both because allergens act via different pathways and because their relationship with the hygiene hypothesis is not yet clear.

### 8.3. The Content of “Hygiene Hypothesis” Could Be Modified

The original statement pointing out “more infections in early childhood protect against later atopy” could be modified into “more non-epithelium-damaging viral infections in the presence of organ-specific non-pathogenic bacteria (or certain bacterial products) in early childhood protect against later atopy” to accommodate most of the exceptions.

## Figures and Tables

**Table 1:** Studies demonstrating the biphasic capacity of lipopolysaccharides (LPS).

Authors	Model	Origin of LPS	LPS Dose and Pathway Used	Allergen or Antigen	Allergen Dose and Pathway Used for Sensitization	Allergen Dose and Pathway Used for Challenging	Protocol	Result	Note
Tulic et al., 2000 [41]	male PVG rat	*Salmonella typhimurium*	50 μg/mL inhaled	OVA	100 μg/mL i.p.	Aerosolized 1% OVA at Day 11 after sensitization	Sensitized rats exposed 1 d before or 1, 2, 4, 6, 8, 10, or 12 d after sensitization A second group of sensitized rats were exposed to LPS 18 h after OVA challenge.	Single aerosol exposure to LPS—1 d, and up to 4 d after i.p. OVA protected against allergy. LPS exposure ≥6, 8, or 10 d after sensitization exacerbated the allergy with cellular influx. Exposure of sensitized rats to LPS on Day 12, 18 h after allergen challenge further potentiated the allergen induced inflammatory cell influx, predominantly due to a 20-fold increase in neutrophil influx, making up ≥80% of the cellular content.	Timing of LPS exposure determines protection or exacerbation of allergy.
Eisenbarth et al., 2002 [43]	BALB/cJ mice and BALB/cAnNCr	*Escherichia coli*	Concomitant use of 100 μg (high dose) or 0.1 μg LPS with OVA in sensitizing period	OVA	100 μg OVA in 50 μL PBS intranasally, or 100 μg OVA in 2 mg Al(OH)_3_ intraperitoneally, with LPS depletion	25 μg OVA intranasally	Sensitized on Days 0, 1 and 2, challenged on Days 14,15,18 and 19, killed on Day 21	Mice exposed to LPS-depleted OVA showed no airway inflammatory responses after challenge; those sensitized with OVA containing low dose LPS demonstrated significant Th2 lung infiltrates; those exposed to PBS or low dose LPS alone did not generate pulmonary inflammation after challenge; those sensitized with OVA containing high dose LPS resulted in a Th1 associated response.	TLR4 signaling Is required for Th2 priming to inhaled antigens, and the dose of LPS during sensitizing period regulates the predominance of Th1 or Th2 response.
Lowe et al., 2015 [44]	Male Dunkin-Hartley guinea pigs (GPs)	LPS source not mentioned	Inhaled 30 μg/mL	OVA	Bil. i.p. injection of OVA,150 μg/mL and Al(OH)_3_ 100 mg/mL normal saline	Sensitized GPs were exposed to inhaled OVA (300 μg/mL) on Day 21.	LPS (30 μg/mL) exposure was by two protocols: 72 and 24 h pre-OVA exposure, 48 h pre-OVA and co-administered with OVA by nebulizer, at rate of 0.3 mL/min for 1 h	LPS exposure 24 h before allergen challenge attenuates the early asthmatic response (EAR), whereas co-administered LPS does not influence the EAR. The addition of a second LPS exposure co-administered with OVA prolonged the EAR. Similarly, LPS exposure 24 h before allergen challenge diminished airway hyperreactivity (AHR) to histamine, whereas co-administered LPS prolonged the AHR	Emphasizing to the timing of LPS application
Langenkamp et al., 2000 [45]	Dendritic cells	*Salmonella abortus equi*. Toxic shock syndrome toxin-1 (TSST-1)	20–100 ng/mL LPS 0.1 or 10 ng/mL TSST-1	TSST-1 as antigen			Pretreatment of dendritic cells with LPS, then after 8 or 48 h, 0.1 or 10 ng/mL TSST-1 was added to culture medium	Soon after LPS stimulation DCs primed strong Th1 responses, but later favored Th2 responses. High dose antigen favored Th1 response, yet low dose antigen favored Th2 response. Exogenous IL12 favored Th1 tendency, yet IL4 favored Th2.	This study explored DC’s priming tendency after LPS pre-stimulation, in response to superantigen TSST-1, which was an inflammatory response, but not allergic response.

d: days.

**Table 2:** Studies demonstrating the protective capacity of LPS.

Authors	Model	Origin of LPS	LPS Dose and Pathway Used	Allergen, Antigen or Stimulus	Allergen Dose and Pathway Used for Sensitization	Allergen Dose and Pathway Used for Challenging	Protocol	Result	Note
Carlsten et al., 2011 [52]	Human of age 7	Home dust	Inhaled from environment	Dog allergen	Inhaled from environment	Inhaled from environment	Correlation study	Endotoxin was associated with decreased risk of sensitization to dog allergen.	HDM was also associated with decreased risk of sensitization to dog allergen, which needs further confirmatory studies. However, in Schuijs’ study below, HDM was noted to induce A20 also, though less pronounced than LPS.
Braun-Fahrlander et al., 2002 [3]	Human of age 6-13	Home dust	Inhaled from environment	unspecified	Inhaled from environment	Inhaled from environment	Correlation study	Endotoxin levels in dust were inversely related to the incidence of hay fever, atopic asthma, and atopic sensitization.	
Schuijs et al., 2015 [10]	Female C57Bl/6 wild-type Human normal bronchial epithelial cells Human epithelial cells from asthma patients	Ultrapure LPS purchased from Invivogen. Strain and species not specified.	A single intranasal injection of 1 μg LPS on Day 14, or i.n 100 ng every other day (starting on Day 14). 100 ng overnight 100 ng every other day for 1 week	HDM	1 μg HDM extracts No sensitization Accurate time point of sensitization could not be traced.	10 μg HDM extracts HDM extracts (dose not mentioned) HDM extracts (dose not mentioned)	Mice sensitized on Day 0 with 1 μg HDM, and challenged on Days 7–11 with 10 μg HDM extracts Cells exposed overnight to 100 ng LPS. After 2 weeks, cells stimulated with HDM extract. cell cultures exposed to 100 ng of LPS for 1 week before stimulation with HDMs	Protective LPS led to decreasing of IL5 and IL13 in mediastinal lymph node cells and downregulation of HDM-induced recruitment of cDCs, with moDCs unaffected. LPS pretreatment reduced allergic cytokines production.	TLR4 signaling in ECs induces attenuators of signaling such as A20.
Ganesh et al., 2014 [53]	BALB/c and DO11.10 mice	*Salmonella enterica serovar typhimurium aroA strain SL 7207*	Intragastric inoculation with 0.5~1 × 10^9^ CFU of whole *S. typhimurium (SL 7207)*	OVA	10 μg OVA + adjuvant, i.p.	intranasal use of 30 μg of OVA	Mice were sensitized with OVA i.p. on Days 7, 8, 9, and 20, infected intragastrically with *S. typhimurium* on Days 0, 7, 20, and 27, challenged on Days 20, 24, 27, 30, and 34 by intranasal administration of of OVA	*S.* *typhimurium* infection in mice results in attenuation of cellular airway inflammation, reduced pathology and mucus production in the lungs, expansion of CD11b^+^Gr1^+^ myeloid cells, with no apparent diversion toward Th1.	This study used whole bacteria for experiment, instead of LPS only.
Rodriguez et al., 2003 [54]	C57BL/6J, BALB/c and C3H/HeJ mice	*Salmonella abortus equi*	LPS at a dose of 20 μg/animal was delivered intravenously concomitantly with a second OVA challenge	OVA	4 μg OVA/1.6 mg aluminum hydroxide	10 μg OVA/50 μL saline intranasally	Mice were immunized on Days 0 and 7, and challenged on Days 14 and 21 intranasally	LPS administration suppresses allergic airway inflammation and cytokine production through a mechanism independent of IL12 or IFNγ. Systemic LPS inhibited airway inflammation. Systemic LPS reduced airway hyperreactivity (AHR).	Thus, systemic LPS displayed protective effect, while local LPS displayed pro-inflammatory effect with neutrophilia reaction.
Lin et al., 2016 [9]	H292 cell line	*Escherichia coli*	0.3 to 30 μg/mL co-culture	polyI:C, HPeV1			LPS pretreatment 2 h before polyI:C or HPeV1 co-culture with H292 cells.	The downstream production of TSLP and IL33 by stimulating H292 cells with polyI:C or HPeV1 was reduced with 30 μg/mL LPS pretreatment, but not 0.3 μg/mL LPS	

**Table 3:** Studies demonstrating the pro-inflammatory capacity of LPS.

Authors	Model	Origin of LPS	LPS Dose and Pathway Used	Allergen or Antigen	Allergen Dose and Pathway Used for Sensitization	Allergen Dose and Pathway Used for Challenging	Protocol	Result	Note
Rittirsch et al., 2008 [67]	C57BL/6 mice C57BL/6 mice with neutrophil depleted by antibody	*Escherichia coli (serotype O111:B4)*	50 μg LPS in 40 μL PBS intratracheally, total 2,550,100 μg	nil	nil	nil	Permeability index checked from bronchoalveolar lavage at 0, 2, 4, 6, 8 h.	Maximal permeability at 50 μg, no different from 100 μg; at 6 h, no different from 8 h. When neutrophils depleted, no permeability change. Pathology includes: interstitial and intraalveolar deposits of neutrophils and fibrin, prominence of alveolar macrophages, and intraalveolar hemorrhage.	The LPS concentration used is 1250 μg/mL, as compared to the 0.3 and 30 μg/mL in cell line model [9], and the total LPS used is 50 μg, as compared with total 100 ng to 1 μg LPS in Schuijs’ study [10].
Eutamene et al., 2005 [70]	Male Wistar rats NCI-H292 human airway epithelial cells	*Pseudomonas* *aeruginosa* *Escherichia* *coli (SO55:B5)*	1 μg LPS per rat via intra-tracheal instillate 2 μg/mL LPS for co-culture	nil	nil	nil	LPS from *P. aeruginosa instilled* in the trachea at a constant rate of 10 μL/min for 15 min. LPS from *E. coli* for 15 and 30 min and 1, 2, 3 and 6 h.	Airway epithelial paracellular permeability was increased **,** Leukocytes number in BAL fluid was sixfold higher, with macrophage, neutrophil and lymphocyte numbers significantly increased. Myosin light chain (MLC) phosphorylation occurs after *E. coli* co-culture, and tight junction permeability increased.	*P. aeruginosa* is a strong pathogen for airway [71], so total amount of LPS used is less, as compared with studies above.
Rojas et al., 2005 [68]	C57BL/6 male mice	*Escherichia coli O111:B6*	Intraperitoneally with 1 mg/kg LPS	nil	nil	nil	Mice were inoculated intraperitoneally with 1 mg/kg of LPS from *E. coli O111:B6.*	Sublethal dose of i.p. LPS to mice caused rapid onset of interstitial pulmonary edema, inflammatory cell accumulation, and deposition of fibronectin and collagen in the lungs.	The scale of mg/kg is sublethal, compared to the protective dose scale of ng/mL to μg/mL.
Yao et al., 2017 [69]	Male C57BL/6J mice male Wistar rats	LPS, source not specified	i.p. LPS at the doses of 8 mg/kg i.p. LPS at the doses of 5 mg/kg	nil	nil	nil	Lung injury in mice and rats were induced by i.p. LPS.	Lung tissues revealed interstitial edema and hemorrhage, alveolar wall thickening, increased infiltration of neutrophils and macrophages in the lung parenchyma and alveolar spaces.	Again, the dose of causing acute lung injury is on the scale of mg/kg.
Taveira da Silva et al., 1993 [72]	Human	*Salmonella minnesota*	i.v. LPS	nil	nil	nil	The patient administered i.v. 1 mg of *S. minnesota* LPS, in sterile water, in an attempt to treat a tumor.	Septic shock syndrome induced, including a high-cardiac-output hypotension, disseminated intravascular coagulation, abnormalities of hepatic and renal function, and non-cardiogenic pulmonary edema.	1 mg of purified LPS is equivalent to 15,000 ng/kg, thousands times higher than the usual dose of 4 ng/kg given to normal volunteers in experimental studies. Endothelial cells are much more sensitive to LPS than epithelial cells, with pg/mL level LPS activating endothelial cells in the presence of blood, compared to the relative resistance of respiratory epithelial cells to μg/mL level LPS [66].
Pugin et al., 1993 [66]	Human umbilical vein endothelial cells (HUVEC)	*Escherichia coli* *0111:B4* *Salmonella* *minnesota*	Incubated with different dilutions of *E. coli 0111:B4 or S. minnesota* wild-type LPS, from 10^−1^ to 10^4^ pg/mL	nil	nil	nil	HUVECs incubated with different dilutions of LPS for 6 h	In the presence of whole blood, 1000-fold less LPS was required to achieve the level of HUVEC activation (assessed by VCAM-1 upregulation) observed with plasma alone.	Endothelial cells are sensitive to ng/mL LPS in the absence of blood, but much more sensitive even to pg/mL LPS in the presence of blood.
Rodriguez et al., 2003 [54]	C57BL/6J, BALB/c and C3H/HeJ mice	*Salmonella abortus equi*	LPS at a dose of 20 μg/animal was delivered intranasally concomitantly with a second OVA challenge	OVA	4 μg OVA/1.6 mg aluminum hydroxide	10 μg OVA/50 μL saline intranasally	Mice were immunized on Days 0 and 7, and challenged on Days14 and 21 intranasally	LPS administration suppresses allergic airway inflammation and cytokine production through a mechanism independent of IL12 or IFNγ Local LPS switched the airway inflammation from eosinophilia to neutrophilia. Local LPS increased AHR by neutrophilic inflammation.	Systemic LPS displayed protective effect, while local LPS displayed pro-inflammatory effect with neutrophilia reaction.
Hammad et al., 2009 [62]	Radiation-induced chimeric *Tlr4*-deficient mice with DCs deficient or ECs-like cells	*Rhodobacter sphaeroides*	10 μg or 100 ng per mouse, in 80 μL PBS, intratracheal	HDM	nil	Intratracheal 100 μg HDM	80 μL PBS intratracheal with HDM and LPS	TLR4 expression on lung structural cells, but not on DCs, is necessary and sufficient for lung DC activation and for priming of effector T helper responses to HDM.	TLR4 triggering on structural cells in the presence of HDM caused production of TSLP, GM-CSF, IL25 and IL33. The absence of TLR4 on structural cells, but not on hematopoietic cells, abolished HDM-driven allergic airway inflammation.

nil: not in list.

**Table 4:** Profile of four major players of the hygiene hypothesis model in the presence of high dose LPS before sensitization.

Cell Type	High Dose LPS	polyI:C or Virus
Epithelial cells	Minimal or no effect [31,46].	Neutral because allergic inflammation due to TLRs pathway activation was blocked by pre-exposure to high dose LPS [9].
Dendritic cells	Basically slight Th1 skewing due to high dose LPS with no IL12/IL4 skewing in the context [43,45] and less Th2-promoting mDC2s [57]. Unstimulated peripheral blood mononuclear cells produced more IL10, IL12 and IFNγ,indicating increased spontaneous production of Th1 and regulatory cytokines [103].	Th1 predominant except RSV infection, which displays Th2 pattern [73,75].
Net result: Th1 predominant ^#^		

^#^ Here what we call high dose is relative to low dose, but not to the extent of toxic dose as in Table 3. High dose LPS has minimal or no effect on epithelial cells, but will skew the DCs slightly toward Th1 response when no IL12/IL4 skewing in the context, and produce less Th2-promoting mDC2s.On the other hand, the Th2-prone allergic inflammation of polyI:C or virus was blocked by pre-exposure to high dose LPS, and polyI:C or virus will stimulate DCs to produce Th1 predominant cytokines with the exception of RSV infection, which displays Th2 pattern. The net result of polyI:C or virus stimulation in the presence of high dose LPS on DCs and epithelial cells would thus be Th1 predominant.

**Table 5:** Profile of four major players of the hygiene hypothesis model in the presence of low dose LPS before sensitization.

Cell Type	Low Dose LPS	polyI:C or Virus
Epithelial cells	Minimal or no effect.	Th2 predominant due to TLR3 pathway activation with production of TSLP, IL33, IL25 etc. [31,32,33,34,35], even slightly enhanced by pre-exposure to low dose LPS [9].
Dendritic cells	Basically slight Th2 skewing due to low dose LPS when no IL12/IL4 skewing in the context [43,45,57].	Th1 or Th2 skewing, depending on the relative stimulatory force between Th2-prone allergic cytokines, such as TSLP and IL33 [112,113], and Th1-prone polyI:C or virus [73,75], except RSV, which displays Th2 pattern.
Net result:Th2 predominant ^#^		

^#^ Here what we call high dose is relative to low dose, but not to the extent of toxic dose as in Table 3. Low dose LPS has minimal or no effect on epithelial cells, but will skew the DCs slightly toward Th2 response when no IL12/IL4 skewing in the context. On the other hand, the Th2-prone allergic inflammation of polyI:C or virus was not blocked, or even slightly enhanced by pre-exposure to low dose LPS. polyI:C or virus will stimulate DCs to produce Th1 predominant cytokines with the exception of RSV infection, which displays Th2 pattern. However, Th2-prone signals coming from epithelial cells, such as TSLP and IL33 will skew the DCs toward Th2. The net result of polyI:C or virus stimulation in the presence of low dose LPS on DCs and epithelial cells would thus be Th2 predominant.
